# Laparoscopic ventral and incisional hernia repair using intraperitoneal onlay mesh with peritoneal bridging

**DOI:** 10.1007/s10029-021-02502-9

**Published:** 2021-09-24

**Authors:** F. Ali, G. Sandblom, A. Wikner, G. Wallin

**Affiliations:** 1 Department of Surgery, Faculty of Medicine and Health, Örebro University Hospital, Örebro University, Örebro, Sweden; 2 Department of Clinical Science and Education Södersjukhuset, Karolinska Institutet, Stockholm, Sweden; 3 Department of Surgery, Södersjukhuset, Stockholm, Sweden

**Keywords:** Laparoscopic surgery, Ventral hernia, IPOM, Peritoneal bridging, Defect closure

## Abstract

**Purpose:**

The aim of this study was to assess the feasibility and safety of a novel IPOM procedure with peritoneal bridging (IPOM-pb) for laparoscopic ventral hernia repair, and to compare the outcomes of this procedure with IPOM with- (IPOM-plus) and IPOM without (sIPOM) defect closure.

**Method:**

A single-centre retrospective study comparing a novel IPOM technique with peritoneal bridging (IPOM-pb) with the two commonly used IPOM techniques, IPOM with defect closure (IPOM-plus) and without defect closure (sIPOM). The intraoperative and postoperative data of patients who underwent laparoscopic IPOM ventral hernia repair were reviewed. Preoperative data, recurrence, and postoperative seroma, surgical site infection, and pain, were compared.

**Results:**

From January 2017 to June 2020, a total of 213 patients underwent laparoscopic ventral and incisional hernia repair with IPOM technique. The mean length and width of the ventral hernia was 4.4 ± 1.8 cm and 3.6 ± 1.4 cm, respectively, and the mean BMI was 30.1 ± 5.2 kg/m^2^. The mean operating time was 67 ± 28 min and was longer for IPOM-pb (71 ± 27 min), less for IPOM-plus (63 ± 28 min), and least for sIPOM (61 ± 26 min). The incidence of early postoperative seroma was least in IPOM-pb (1/98, 1%), and similar in the IPOM-plus (4/94, 4%) and sIPOM (1/21, 5%) group. Late postoperative seroma was found only in IPOM-plus (2, 2%). The incidence of early and late postoperative pain was relatively higher in sIPOM (3, 14%; 1, 5%, respectively) compared to IPOM-pb and IPOM-plus in the early (5, 5% and 6, 6%) and late (2, 2% and 1, 1%) postoperative period, respectively. Surgical site infection was higher in sIPOM group (3, 14%), compared to IPOM-pb (1, 1%), and IPOM-plus (3, 3%). Recurrence rates were similar in IPOM-pb group (3/98, 3%) and IPOM-plus (3/94, 3%), and none in sIPOM (0/21).

**Conclusion:**

IPOM with peritoneal bridging is as feasible and safe as conventional IPOM with defect closure and simple non-defect closure. However, a large randomised controlled trial is required to confirm this finding.

## Introduction

The life-time risk for developing a ventral hernia has been estimated at 5% in the general population [1, 2]. Ventral hernias are either primary, or secondary to abdominal surgery (incisional). The cumulative incidence of incisional hernia may be as high as 28% following open abdominal surgery [1, 3–5]. Since it was introduced by Karl Leblanc [6] in 1993, laparoscopic ventral hernia repair (LVHR) has gained increasing acceptance due to better postoperative outcomes compared to open ventral hernia repair (OVHR) [7–10], but there is considerable controversy regarding the optimal approach. Two laparoscopic approaches are commonly used in LVHR: simple intraperitoneal onlay mesh (sIPOM); and IPOM with defect closure prior to placement of mesh (IPOM-plus).

Seroma formation is a common complication after LVHR, resulting in poor aesthetic outcome, discomfort, pain and infection [11, 12]. Seroma rates after LVHR vary greatly, and those detected by clinical examination alone have been previously reported to range from 0.5% to 35% [13].

Various techniques have been suggested to reduce seroma formation in the hernial sac anterior to the mesh, but only conventional defect closure in the IPOM-plus procedure seems to reduce postoperative seroma formation significantly [12]. One possible explanation for the lower seroma rate after defect closure is because of the reduced dead space in the residual hernial sac. On the other hand, it is claimed that surgical tension created by defect closure in the IPOM-plus procedure may result in more postoperative pain, discomfort, and/or fatigue [1, 12, 14–16].

Instead of the IPOM-plus approach where the hernial defect is closed by suture, part of the peritoneum can be dissected up to the midpoint of the hernial sac to create a peritoneal flap that is subsequently used to bring down the hernial sac and suture it intra-abdominally prior to mesh application. Compared to conventional IPOM-plus, this IPOM-peritoneal bridging approach could theoretically lead to reduced postoperative seroma formation due to eradication of the dead space otherwise created by the hernial sac. Furthermore, avoidance of surgical tension created by suturing the hernial defect should reduce postoperative pain, discomfort, and/or fatigue. The benefits of less mesh bulging and recurrence rates due to the greater intra-abdominal attachment area for mesh application after hernial defect closure are maintained [1, 14, 17].

The aim of this study was to assess the feasibility and safety of a novel IPOM procedure with peritoneal bridging (IPOM-pb) for laparoscopic ventral hernia repair, and to compare the outcomes of this procedure with two commonly used procedures, IPOM with- (IPOM-plus) and IPOM without (sIPOM) defect closure.

## Method

Data for this study were retrospectively obtained by identifying patients who underwent LVHR between January 2017 and June 2020 and extracting pre-, per-, and postoperative data from the local medical records at Karlskoga Hospital, Orebro county, Sweden. The study protocol was approved by the Ethics Review Board of Uppsala University (EPN Dnr 2020–03,259).

The database included outcomes following LVHRs during the study period, including simple IPOM (sIPOM), conventional IPOM with defect closure (IPOM-plus), and IPOM-peritoneal bridging (IPOM-pb). The decision of surgical method was taken by the surgeon in dialogue with the patient and was based on the surgeon’s own clinical assessment in each respective case. Data included patient demographics (age, gender, body mass index (BMI), hernia characteristics (aetiology, localisation, dimensions), procedure-related data (start-to-finish operating time, intraoperative events/complications), and postoperative outcomes (surgical site infection, seroma, pain/discomfort, and recurrence). Hernia characteristics were divided into epigastric, umbilical, and incisional hernia. Presence of seroma was dichotomised as either present or absent. Postoperative pain was estimated using a VAS score from 1 to 10. The criterium for inclusion was ventral hernia undergoing repair with laparoscopic IPOM-pb, IPOM-plus, or sIPOM technique.

## Comparisons between the IPOM techniques

**Fig. 1:**
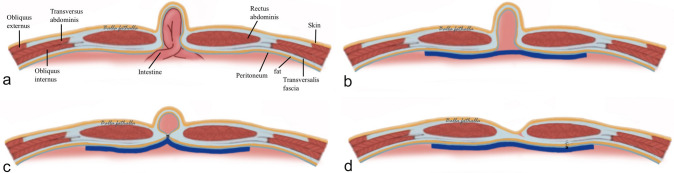
Schematics of ventral hernia (**a**) and final result following hernia repair with sIPOM (**b**), IPOM-plus (**c**), and IPOM-pb (**d**)

The difference between sIPOM, IPOM-plus and IPOM-pb techniques is the way the hernia defect and the sac are handled (Fig. 1a–d). In sIPOM, the mesh placement is done without suturing the hernial defect (Fig. 1b). In IPOM-plus, the hernial defect is sutured prior to mesh placement (Fig. 1c). In sIPOM as well as IPOM-plus, the hernial sac is left in situ. In IPOM-pb, peritoneal bridging is done to eliminate the hernial defect and the sac, thereby closing the cavity of hernial sac prior to mesh placement (Fig. 1d). The peritoneal bridging is achieved by creating a peritoneal flap halfway into the hernial sac that is then brought down together with the hernial sac and bridged across the defect and sutured (described in more detail in step one and two below).

In all three IPOM techniques, the mesh placement was achieved using absorbable tacks (Absorbatack^™^) as described in step three below. In IPOM-plus and IPOM-pb, the material used for suturing the defect and for suturing the peritoneal flap, respectively, was PDS^®^ 2–0 (Stratafix^™^) suture. Except for step one and two, which specifically describes the peritoneal bridging technique, all other steps described below was also done for all three IPOM techniques.

## Surgical technique

### Patient preparation

**Fig. 2:**
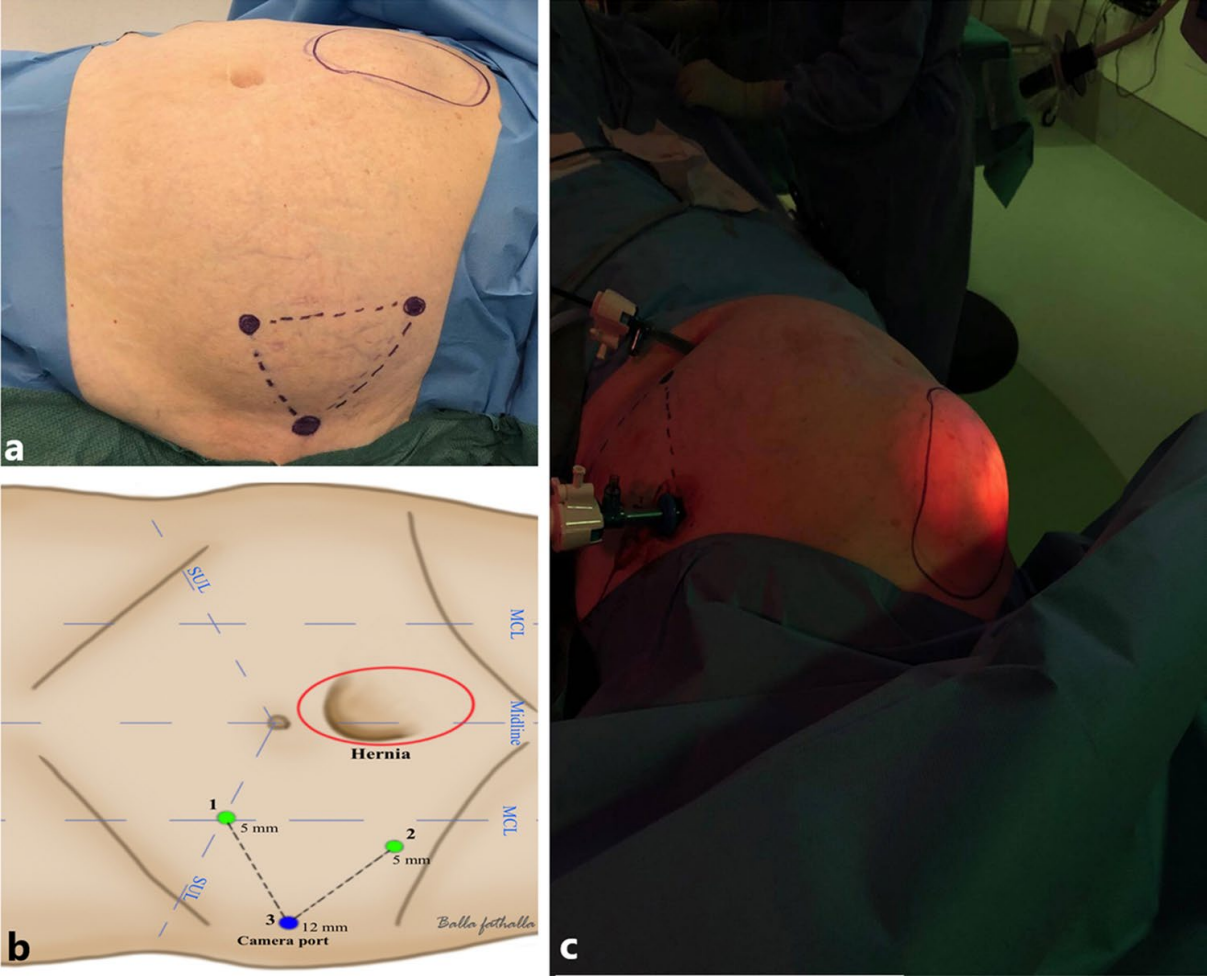
Anatomical landmark (**a, b**) and tilting positioning of the patient (**c**) to optimise surgeons manoeuvrability

Patients undergoing IPOM-pb underwent the same preoperative preparations as all other patients undergoing LVHR according to standard operative protocol. The anatomical landmarks identifying the hernia border marked on the abdominal wall (Fig. 2a, b). The procedure was performed under general anaesthesia. The patient was in the supine position with both arms at 90° to the side to provide maximum room for the surgeon and operation assistant (Fig. 2c).

### Adhesiolysis and reduction of hernial content

**Fig. 3:**
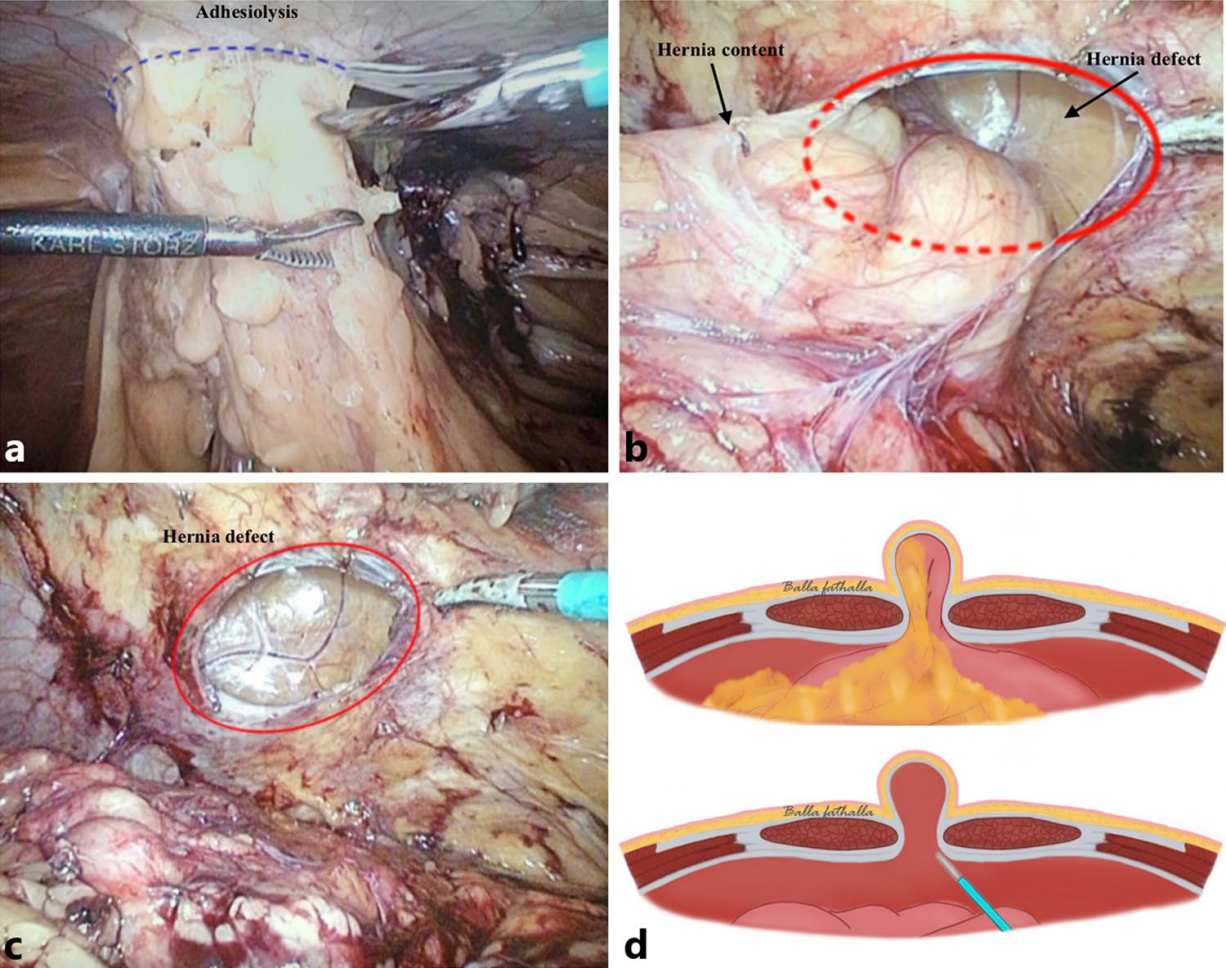
Adhesiolysis (**a**) and reduction of hernia content (**b, c**). The schematics (**d**) illustrate this adhesiolysis and hernia reduction step

Using the laparoscopic grasper, adherences were grasped and adhesiolysis carried out, preferably with cold scissors (Fig. 3a). Use of diathermy was restricted to avoid thermal injury. Adhesiolysis continued until all adhesions around the hernia defect had been released (Fig. 3a–b). Reduction of the intestinal content was achieved by gentle manipulation using the laparoscopic grasper while the operation assistant gently pressed on the hernia from the outside of the abdominal wall (Fig. 3b–c). The reduced part of the viscera was inspected to rule out ongoing bleeding.

### Step one: creation of a peritoneal flap

**Fig. 4:**
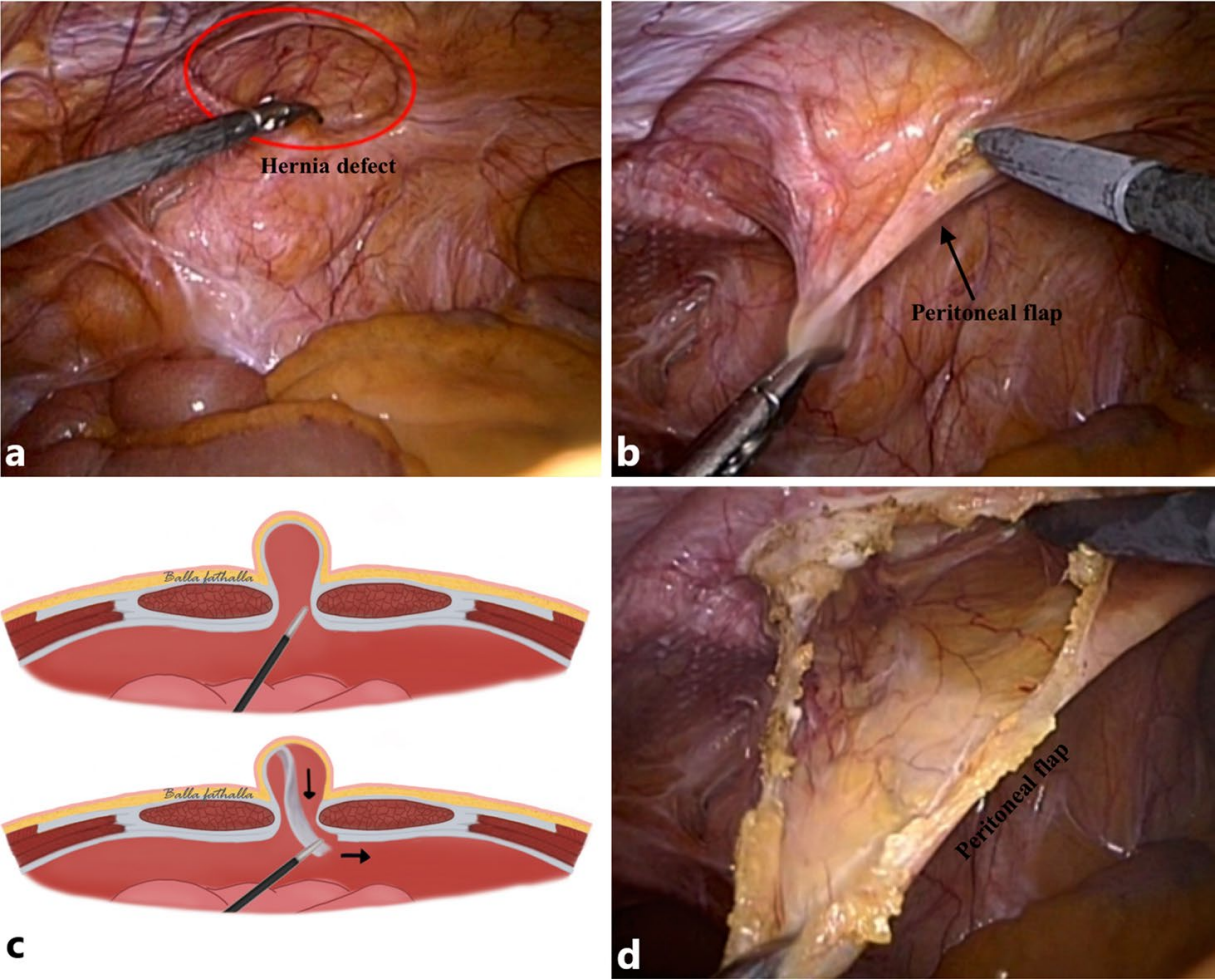
The top photographs shows that the hernia defect is grasped in the middle (**a**) and retracted (**b**) to enable dissection with diathermy in order to create a peritoneal flap (**d**). The same step is illustrated in the schematic (**c**)

The peritoneum was grasped in the middle of the hernial sac (Fig. 4a, b) and retracted so that the area approximately 1.5–2 cm from the hernial defect margin was isolated, enabling dissection of the margin using monopolar diathermy (Fig. 4b). The free part of the peritoneum was dissected until the midline of the hernial sac was reached to create a peritoneal flap (Fig. 4c, d).

### Step two: peritoneal bridging and suturing of the peritoneal flap

**Fig. 5:**
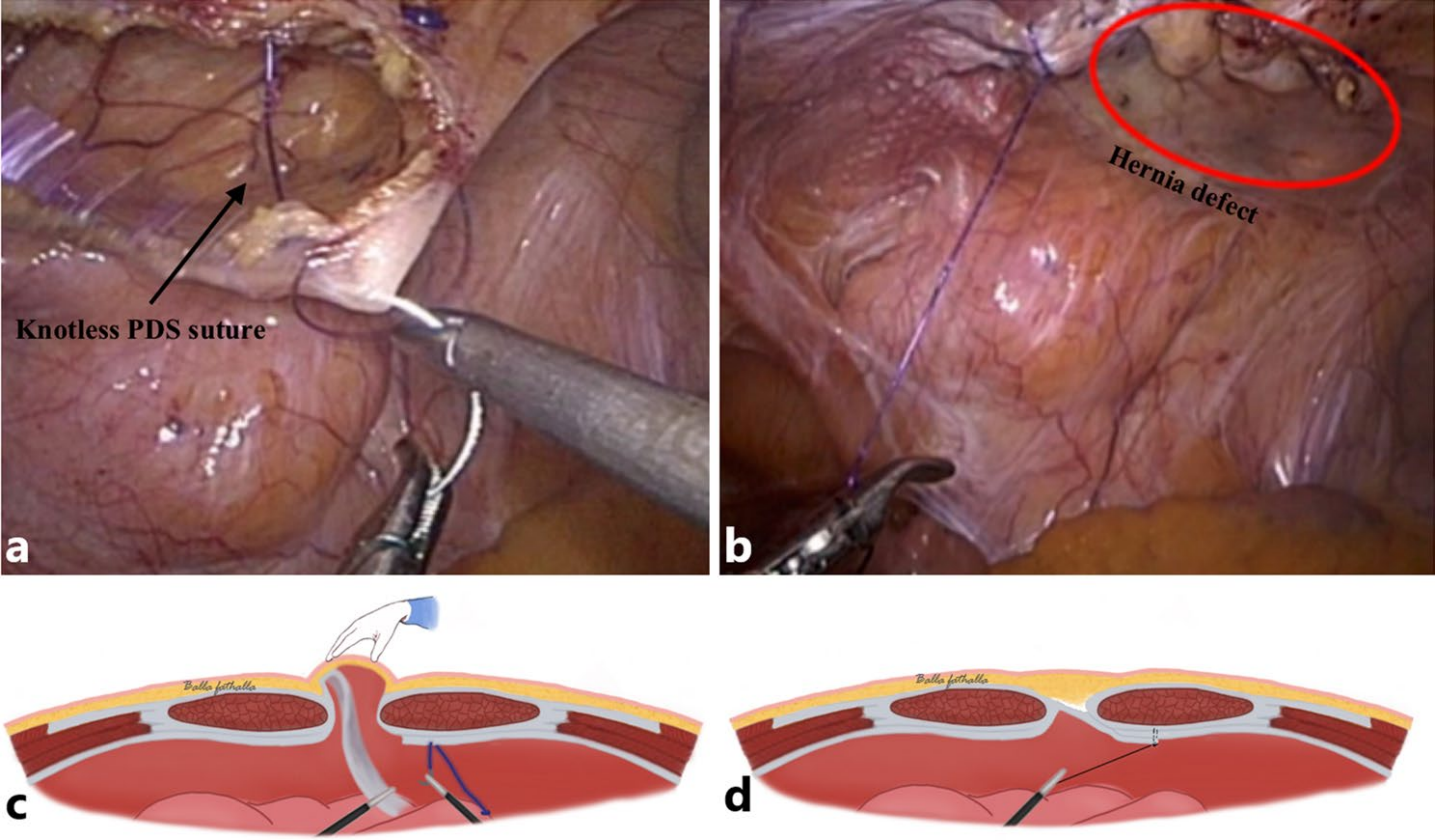
The peritoneal flap is pulled down while the assistant pushes the hernial sac to minimise the dead space (**a, c**), which is then sutured (**b, d**)

The peritoneal flap was then pulled down while the assistant pressed the hernia from the outside to diminish the dead space of the hernial sac cavity (Fig. 5c). The peritoneal flap was then pulled to the initial dissected site and fixed with knotless PDS^®^ 2–0 (Stratafix^™^) to the edge of the aponeurosis (Fig. 5a–d).

### Step three: mesh placement and finishing procedures

The mesh used was polypropylene Ventra light^®^ ST with ECHO 2^®^ positioning system mesh. This is a low-profile, bioresorbable, permanent mesh weighing 51 g/m^2^. It has a pre-attached removable positioning system and is coated with dual components (absorbable and non-absorbable).

**Fig. 6:**
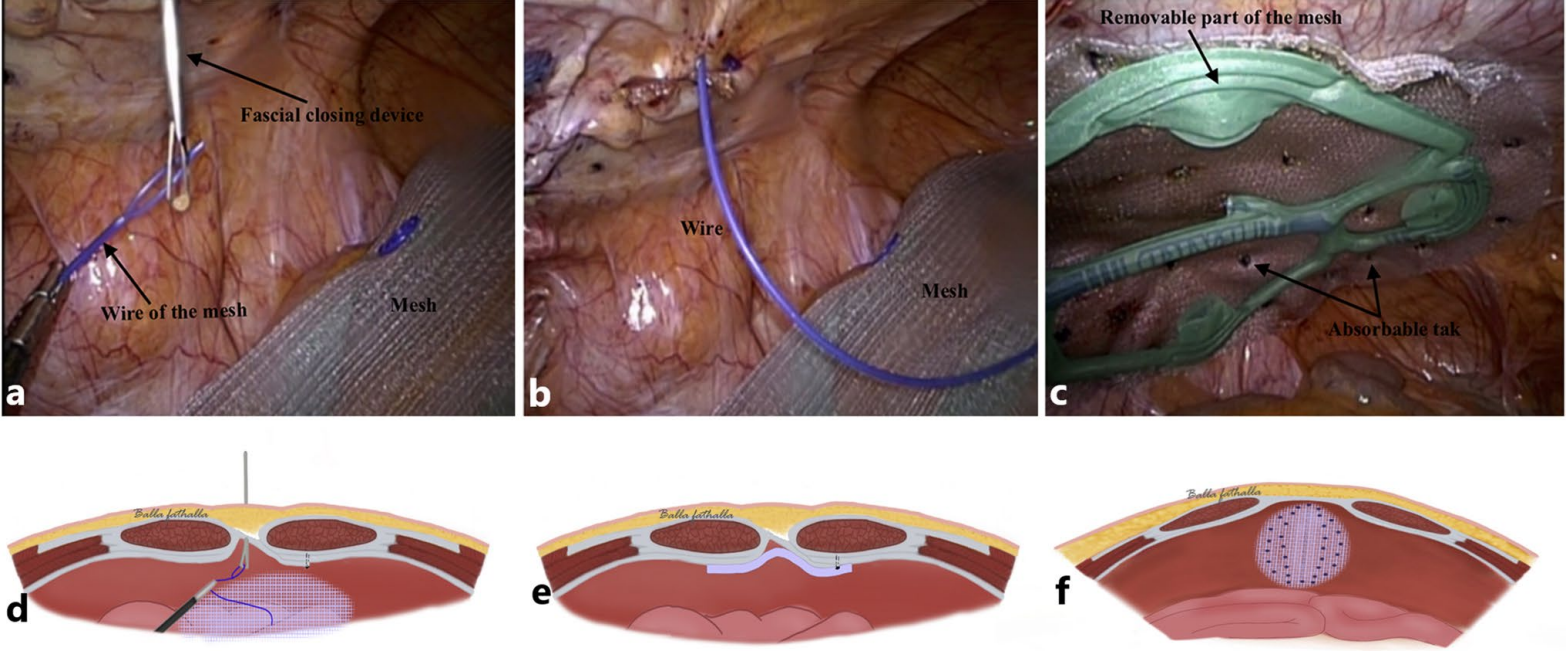
The fascial closure device is introduced under laparoscopic vision (**a, d**) and the mesh wire is pulled out of abdomen (**b,e**). The mesh is subsequently fixated using a double-crown technique (**c, f**)

The fascial closure device was introduced through the midline of the hernia defect under laparoscopic vision (Fig. 6a, d). The mesh wire was grasped with the fascial closure device and pulled out of the abdomen (Fig. 6a–c, d–f), causing the mesh to have complete contact with the intra-abdominal surface of the abdominal wall.

The laparoscopic grasper was subsequently used to manipulate the mesh to ensure 5 cm overlap of the hernia defect margin. The mesh was fixated 1 cm from the margin with 2 cm between each fixation point, with absorbable tacks (Absorbatack^™^) using a double-crown technique (Fig. 6c, f). Lastly, the belt of the mesh (*i.e.* the positioning system) was removed from the abdomen through the 5 mm port.

The procedure was completed by exploring the abdominal cavity for bleeding and intestinal injury before removing the trochars and completing surgery. The fascia of the camera port was sutured with PDS^®^ 2–0. The cutis of the camera port and the two working ports was sutured with 3–0 absorbable suture intracutaneously.

## Postoperative course

Most patients who underwent LVHR were discharged the same day. Patients who required overnight stay were mostly those with comorbidities that required monitoring after general anaesthesia. All patients received an elastic abdominal girdle and were instructed to wear it continuously for 14 days after index surgery, followed by daytime use 14 days thereafter.

## Statistical analyses

Categorical variables were presented as numbers (percentages) and continuous variables as either mean (standard deviation, SD) or median (interquartile range, IQR). The size of the mesh was not routinely reported, but the principle of at least 5 cm mesh overlap was followed. The mesh-to-defect-ratio (MDAR) was calculated using the formula as given by Tulloh and de Beaux [18], and the mesh size was calculated based on the assumption that the principle of 5 cm mesh overlap was followed (i.e. 5 cm mesh overlapping the defect on all directions). Statistical assessments, including the one-minus survival function analysis, were performed using SPSS (Statistical Package for Social Sciences for Windows, version 25.0, Armonk, NY, USA: IBM Corp.).

## Results

**Fig. 7:**
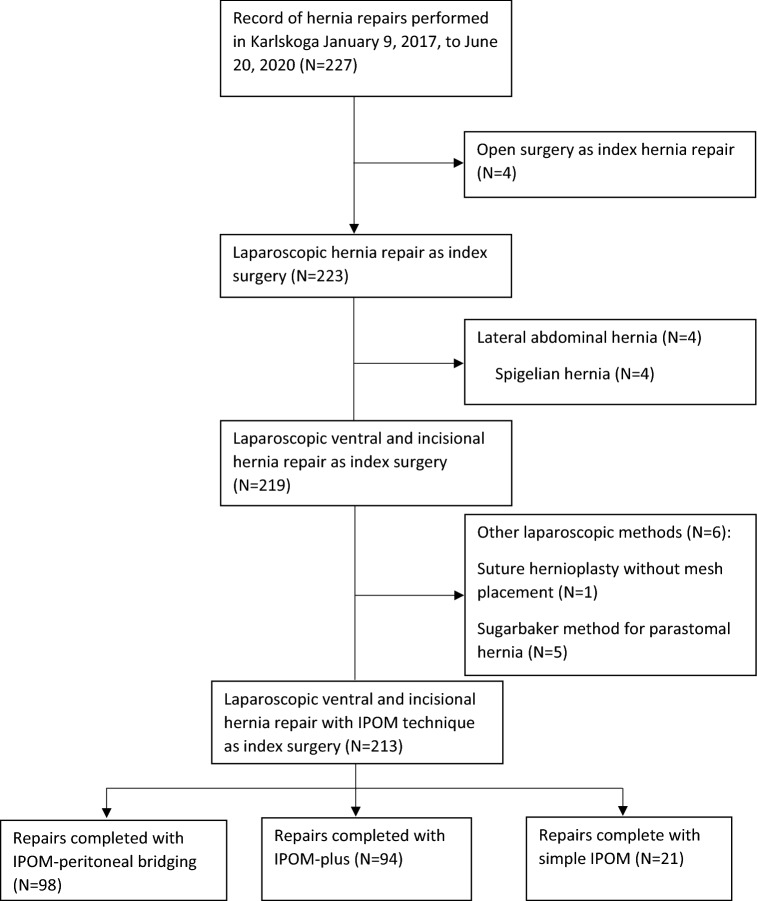
Flow chart

A total of 227 patients who underwent abdominal hernia repair were identified in the local medical records. A total of 14 patients were excluded: four patients had open surgery, four patients had Spigelian (i.e. lateral) hernia, one patient underwent laparoscopic suture-only hernioplasty with no mesh placement and five patients with parastomal hernia had laparoscopic hernia repair by Sugarbaker technique (Fig. 7). These patients did not meet the inclusion criteria of undergoing laparoscopic ventral and incisional hernia repair with IPOM method and were thus excluded from analysis. Patients whose operation was converted to open surgery remained in the study and were analysed according to intention to treat. This left 213 patients for evaluation of whom 21 (10%) was in simple IPOM (sIPOM), 94 (44%) was in IPOM with defect closure (IPOM-plus), and 98 (46%) was in IPOM with peritoneal bridging (IPOM-pb) group. Of the 213 patients, 12 underwent urgent or emergent surgery and 201 patients underwent elective surgery performed by three surgeons at Karlskoga Hospital, Sweden.

Patient demographics and type of hernia as reported in the local medical record are presented in Table 1. Gender distribution were similar in the three groups. The patients in the sIPOM group were on average slightly younger and had less BMI than patients in the IPOM-pb and IPOM-plus group. Cardiovascular comorbidity was slightly more prevalent in the IPOM-pb group, and there were higher percentage of active smokers and of patients with chronic obstructive lung disease (COPD) in the sIPOM group than the other three groups. ASA class and types of hernia were similar in all groups except for that there was no epigastric hernia in the sIPOM group.

**Table 1:** Baseline characteristics

	IPOM-pb (*N* = 98)	IPOM-plus (*N* = 94)	sIPOM (*N* = 21)	All repairs (*N* = 213)
Gender				
Male	49 (50%)	48 (51%)	10 (48%)	107 (50%)
Female	49 (50%)	46 (49%)	11 (52%)	106 (50%)
Age, years, mean (SD)	60 (15)	58 (15)	55 (11)	59 (14)
BMI, kg/m^2^, mean (SD)	30.6 (5.6)	30.0 (4.6)	28.3 (5.4)	30,1 (5,2)
Cardiovascular comorbidities*, *n* (%)	26 (27%)	20 (21%)	4 (19%)	50 (23%)
COPD, *n* (%)	3 (3%)	5 (5%)	2 (10%)	10 (5%)
Smoking, *n* (%)	10 (10%)	11 (12%)	4 (19%)	25 (12%)
Diabetes, *n* (%)	9 (9%)	8 (9%)	2 (10%)	19 (9%)
ASA score**				
ASA-I, *n* (%)	28 (29%)	27 (29%)	1 (5%)	56 (26%)
ASA-II, *n* (%)	57 (58%)	54 (57%)	17 (81%)	128 (60%)
ASA-III, *n* (%)	13 (13%)	13 (14%)	3 (14%)	29 (14%)
Type of hernia				
Epigastrical	8 (8%)	9 (10%)	0 (0%)	17 (8%)
Umbilical	37 (38%)	34 (36%)	8 (38%)	79 (37%)
Incisional	53 (54%)	51 (54%)	13 (62%)	117 (55%)

^*^DVT, MI, LE, and/or atrial fibrillation in the patient history

^**^Number of patients (percentage of ASA score in each respective group

The peroperative and postoperative outcome measures are presented in Table 2. The median follow-up time was at least six months shorter for IPOM-plus than for simple IPOM and IPOM-peritoneal bridging.

**Table 2:** Outcome measures

	Peritoneal bridging (*N* = 98)	IPOM-plus (*N* = 94)	Simple IPOM (*N* = 21)	All repairs (*N* = 213)
Thromboprophylaxis	10 (10%)	6 (6%)	2 (10%)	18 (8%)
Antibiotic prophylaxis	57 (58%)	63 (67%)	3 (14%)	123 (58%)
Peroperative data				
Operative time, minutes, mean (SD)	71 (27)	63 (28)	61 (26)	67 (28)
Vertical length, cm, mean (SD)^a^	4.7 (1.7)	4.5 (1.7)	3.1 (1.8)	4.4 (1.8)
Horizontal width, cm, mean (SD)^a^	4.0 (1.2)	3.6 (1.4)	2.0 (0.9)	3.6 (1.4)
Mesh:defect area ratio, median (IQR)	9 (7–19)	9 (7–19)	36 (12–36)	12 (7–19)
Conversion to open surgery	0 (0%)	1 (1%)	1^*^ (5%)	2 (1%)
Bleeding	2 (2%)	0 (0%)	0 (0%)	2 (0.5%)
Intestinal injury	0 (0%)	1 (1%)	1^*^ (5%)	2 (1%)
Postoperative complication				
Surgical site infection	1 (1%)	3 (3%)	3 (14%)	7 (3%)
Seroma (≤ 1 month)	1 (1%)	4 (4%)	1 (5%)	6 (3%)
Seroma (≥ 6 months)	0 (0%)	2 (2%)	0 (0%)	2 (1%)
Postoperative pain^c^ (≤ 1 month)	5 (5%)	6 (6%)	3 (14%)	14 (7%)
Postoperative pain^c^ (≥ 6 months)	2 (2%)	1 (1%)	1 (5%)	4 (2%)
Recurrence	3 (3%)	3 (3%)	0 (0%)	6 (3%)
Worsening cardiovascular Condition	0 (0%)	1^b^ (1%)	1^*^ (5%)	2 (1%)
Deaths^*^^*^	1 (1%)	2 (2%)	1^*^ (5%)	4 (2%)
Median follow-up, months (IQR)	27 (13–34)	20 (10–31)	26 (19–34)	24 (11–33)

^a^Of the 213 patient records, the width was not reported in 48% of cases. In these cases the vertical length was presumably meant as diameter length. The vertical length and width in this table is noted as it was reported in the local medical record

^b^The patient desaturated after index IPOM-plus and after further investigation was diagnosed with lung sarcoidosis

^c^Postoperative pain requiring analgesia

^*^The patient with planned laparoscopic sIPOM had extensive adhesions that necessitated conversion to open surgery. The patient deteriorated postoperatively, suspected to have intestinal injury, multiple laparotomies and VAC therapy because of intestinal injury. The patient died 3 months after index surgery

^**^The reported cause of death in IPOM-pb and IPOM-plus was unrelated with their respective index surgery. Numbers are in absolute values (percentage), unless stated otherwise

The mean operating time for IPOM-pb was 8 min longer than IPOM-plus, and 10 min longer than sIPOM (71 ± 27 min *vs* 63 ± 28 min *vs* 61 ± 26 min, respectively). The median length and width were at least 1.4 cm smaller, respectively, in sIPOM compared to IPOM-plus and IPOM-pb. The median mesh-to-defect-area ratio (MDAR) was four times larger in sIPOM compared to IPOM-plus and IPOM-pb.

Four patients were reported deceased in the medical records in total: one patient in IPOM-pb 18 months after operation, two patients in IPOM-plus 5- and 15 months after respective operation, and one patient in sIPOM 3 months after operation. The reported primary cause of death in IPOM-pb and IPOM-plus group was unrelated to the index hernia repair surgery. In the sIPOM group, the patient had previous to index surgery multiple abdominal surgeries, multiple events of abdominal abscesses and chronic portal venous thrombosis. On the index surgery, the patient had extensive adhesions which necessitated conversion to open surgery. After the index surgery, the patient deteriorated postoperatively, which raised suspicion of intestinal injury. The patient was admitted to Örebro University Hospital, where he underwent multiple explorative laparotomies because of intestinal injury, small bowel resections and vacuum-assisted closure therapy. The patient died three months after index surgery.

Apart from the aforementioned patient in sIPOM group, another patient in the IPOM-plus group was reported to have worsening condition in the form of desaturation postoperatively, computed tomography was done for signs of lung emboli that showed signs of atelectasis and enlarged mediastinal lymph nodes: the patient received final diagnosis of lung sarcoidosis. No cases of worsening cardiovascular condition were reported in IPOM-pb group.

The incidence of early postoperative seroma was lowest in the IPOM-pb group (1/98, 1%), and slightly less in IPOM-plus (4/94, 4%) and highest in sIPOM (1/21, 5%) group. Late postoperative seroma was found only in the IPOM-plus group (2, 2%). The incidence of early and late postoperative pain was relatively higher in sIPOM (3, 14%; 1, 5%, respectively) compared to IPOM-pb and IPOM-plus in the early (5, 5% and 6, 6%) and late (2, 2% and 1, 1%) postoperative period, respectively. Surgical site infection rate was higher in sIPOM group (3, 14%), compared with the IPOM-pb (1, 1%), and IPOM-plus groups (3, 3%).

Three patients had recurrence after IPOM-pb, three patients after IPOM-plus, and none in sIPOM group. For the IPOM-pb group, two patients developed recurrence after 18 and 26 months. Both were verified by CT abdomen. For the third patient the medical journal was unclear but was reported to have recurrent hernia 24 months after index surgery at a hospital in another region with a different medical record system. For the IPOM-plus group, one patient was reported to have recurrence by CT abdomen 6 months after index surgery and two patients were reported by clinical examination to have recurrence 8 and 20 months after surgery.

**Fig. 8:**
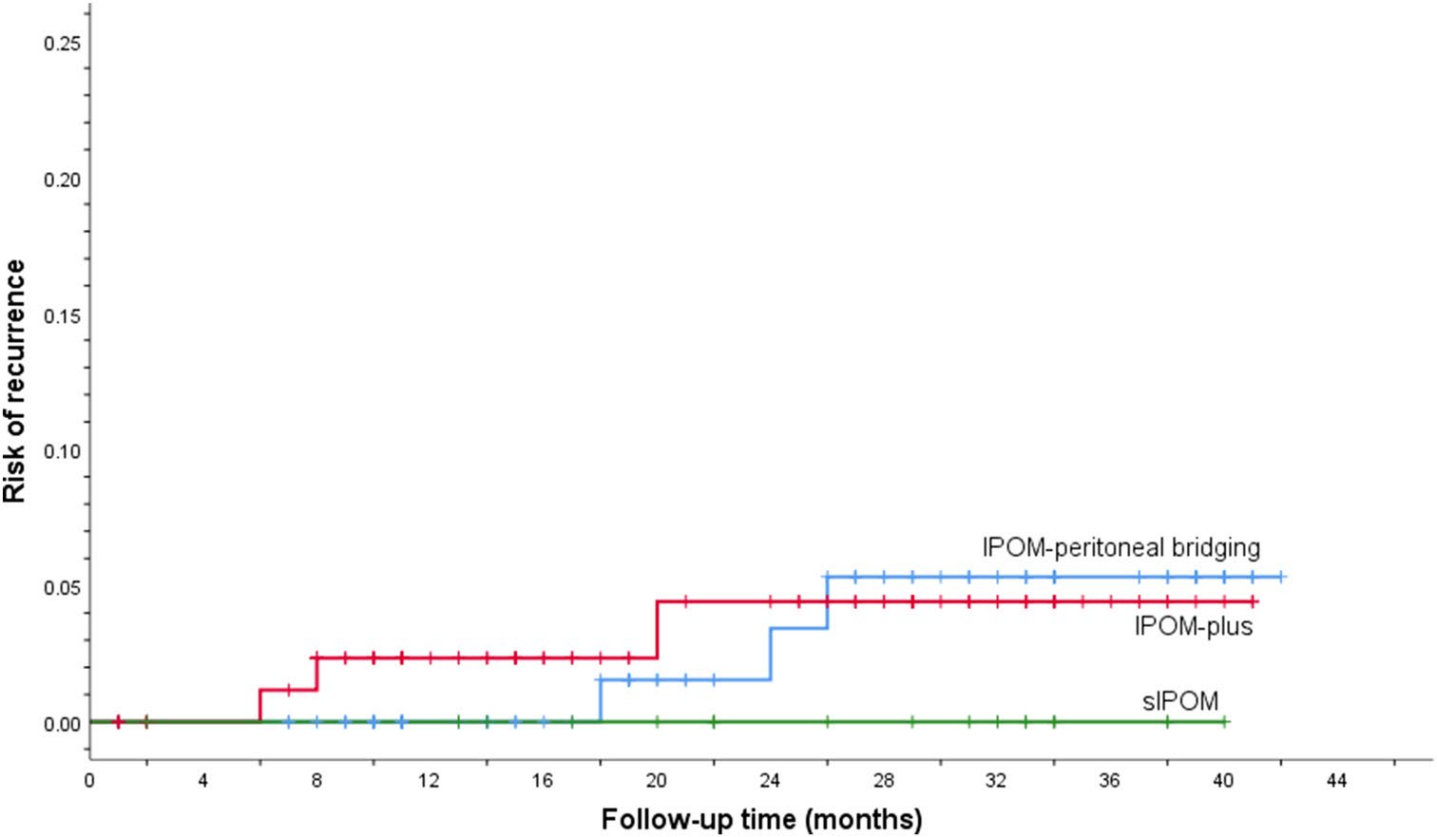
The cumulative recurrence rate for sIPOM, IPOM-plus and IPOM-pb, respectively

Recurrences for each IPOM technique is shown in Table 2. The time-to-event analysis for hernia recurrence is presented as one-minus survival function with Kaplan–Meier plot in Fig. 8.

## Discussion

The present study shows that IPOM-pb repair is safe and may be used for routine LVHR. The technique does not require additional resources or extra efforts from the theatre staff or surgeon. Further studies are required to evaluate the potential benefits of the technique.

Laparoscopic ventral hernia repair (LVHR) has increasingly gained acceptance since introduction in 1993 [6] because of its favourable outcome compared to open ventral hernia repair (OVHR) [7–10], but there is considerable controversy regarding the optimal approach. At present, two laparoscopic approaches are commonly used: simple intraperitoneal onlay mesh (sIPOM) repair and IPOM with defect closure prior to mesh placement (IPOM-plus). The aim of this study was to evaluate a novel IPOM procedure with peritoneal bridging (IPOM-pb) by retrospectively reviewing the medical records of patients who underwent surgery with any of the three IPOM approaches at a single centre between January 2017 and June 2020.

There are possible technical challenges to take into consideration before embarking on IPOM-pb. It may be difficult to free the peritoneal flap in some patients with thinning/atrophy of the skin covering the hernia sac, that may have been resulted from a longstanding pressure from hernial content on the skin. A longstanding hernia may cause thinning and/or necrosis of the skin, which may increase the risk for perforation or surgical site infection. In this situation, open ventral hernia repair with resection of the skin may be preferable. Multiple smaller hernia defects may also pose a technical challenge or prolong operation time when freeing the flap in the peritoneal bridging procedure. The advantages of the peritoneal bridging technique are greater with a large single defect than with multiple smaller hernia defects.

Furthermore, challenges facing the surgeon following ventral hernia repair include recurrence, seroma formation, pain/discomfort, and surgical site infection [12].

One possible advantage of defect closure (by suture or peritoneal bridging, i.e. IPOM-plus or IPOM-pb) prior to mesh placement is that it increases the attachment area for the mesh, which hypothetically would result in lower recurrence rate [18–20]. A recent study by Christoffersen et al.compared outcomes between IPOM-plus with sIPOM, defining recurrence rates by the findings at clinical assessment and/or reoperation, and if inconclusive by CT abdomen [14]. The incidence of recurrence was nearly three times lower in the IPOM-plus group compared to the sIPOM (5/36, 14% vs 12/37, 32%) (p = 0.047). In contrast, another study by Bernardi et al. [17] reported recurrence rates two years after surgery, confirmed clinically or with CT abdomen, to be higher following IPOM-plus than sIPOM (6/61; 9,8% vs 2/62; 3,2%, *p* = 0.131). Similarly, the data in the present retrospective study showed lower recurrence rate after sIPOM (0/21) compared to IPOM-plus, whereas the recurrence rate were similar for IPOM-plus and IPOM-pb (3/98, 3% vs 3/94, 3%) (Table 2). The cumulative incidence of recurrence was higher in the IPOM-pb group compared to the IPOM-plus (Fig. 8). The lower BMI in sIPOM compared to the other two IPOM methods may have contributed to this result. Another possible factor that may have contributed to the lower recurrence rate in sIPOM is the smaller defect size, which for sIPOM was on average at least 1.4 cm smaller in length and width, respectively, compared to IPOM-plus and IPOM-pb (Table 1). A recent systematic review by Parker *et.al* found that a wider defect appeared to increasingly predispose to higher recurrence [21].

Furthermore, a higher mesh-to-defect-area ratio (MDAR) have been reported in previous literature as an additional potential factor for lower risk of recurrence [18–20]. In this study, the MDAR was four times higher in sIPOM compared to IPOM-plus and IPOM-pb, respectively. This means that the forces resisting mesh displacement are four times stronger [18], which could further explain the lower recurrence rate in sIPOM compared to IPOM-plus and IPOM-pb.

Although nearly all of the patients may have seroma formation anterior to the mesh in the early postoperative period after LVHR [13, 22], they tend to be often asymptomatic and spontaneously resolving. However, seroma as a complication after LVHR is still a common complication that may lead to poor aesthetic outcome, discomfort, pain or surgical site infection [11, 12]. Although the cause of seroma formation is still largely unknown, previous studies suggest that elimination of the dead space caused by the residual hernia sac may lead to significantly less postoperative seroma formation [12, 23].

However, the dead space cannot be eliminated by simply excising the residual hernia sac as entirely freeing the hernia sac from the overlying skin laparoscopically is technically challenging and would require more operative time. Nevertheless, the benefit may even be minimal as the dead space may still remain between the overlying skin and the mesh that could potentially still result in seroma formation, and the issue of abdominal wall weakness between the mesh and the skin would still have to addressed. For this reason, several other methods have been suggested that aim at reducing or eliminating the residual hernia sac, but only conventional defect closure in the IPOM-plus technique seems to reduce postoperative seroma significantly [12, 23]. In a recent 1-month follow-up study by Christoffersen et al. [14], seroma was assessed by clinical examination and, if inconclusive, by abdominal CT. Seroma was found in 58% of patients after sIPOM and 25% after IPOM-plus repair. The lower incidence of seroma after defect closure could be explained by the smaller dead space in the residual hernia sac.

Furthermore, since peritoneal bridging further eliminates the dead space by not leaving the hernia sac in situ, even less postoperative seroma is expected in IPOM-pb. In this study, the incidence of seroma within 1 month after index surgery was highest in sIPOM (1/21, 5%), less in IPOM-plus (4/94, 4%), and least in IPOM-pb (1/98, 1%). Nevertheless, the postoperative incidence of seroma 6 months or more after index surgery was only reported in the IPOM-plus (2/94, 2%) and none in sIPOM or IPOM-pb. This discrepancy in the results may be explained by the smaller number of patients in sIPOM group compared to IPOM-plus and IPOM-pb.

Furthermore, it is claimed that the surgical tension created by defect closure in IPOM-plus repair may result in more postoperative pain, discomfort, or fatigue [1, 12, 14–16]. The aim of the peritoneal bridging approach is to improve overall outcome by eradication of the dead space while preserving the tension-free surgery principle. In the present study, the percentage of patients requiring analgesia after surgery was nearly twice as high in the non-defect closure group compared to the peritoneal bridging and defect closure groups. However, due to the nature of this retrospective review, the variables in the study could not be controlled, there may, thus, have been confounding effects of previous or postoperative medical conditions requiring analgesia that influenced the result. Additionally, the number of patients in the sIPOM was at least four times smaller than the other two groups, in which a small incremental increase in absolute number causes a relatively greater incidence that may have contributed to the higher percentage of postoperative pain.

The mean operating time was slightly longer in the peritoneal bridging group compared to the IPOM-plus and sIPOM group as a result of the additional steps prior to mesh placement. The postoperative surgical site infection was more than three times higher in sIPOM group compared to IPOM-pb and IPOM-plus. The higher rate of surgical site infection in sIPOM could be explained by that only 14% of patient received prophylactic antibiotics in sIPOM compared to over half of the patients in IPOM-pb and IPOM-plus, respectively.

This study has several limitations. First, since this was a retrospective review, the data collected from the local medical record system depended on the limited information provided by the surgeon performing the procedure and the physician on call, and varied in detail and reliability. Second, the study was not double-blinded, randomised, or controlled, and was thus subject to bias and possible confounding factors that may have influenced the results. Until now, there has only been one randomised controlled trial [15] evaluating a total of 50 patients (25:25) who underwent peritoneal bridging (not to be confused with simple IPOM) and conventional defect closure. Apart from acute pain one week after index surgery being slightly higher in the defect closure group, seroma, surgical site infection, and recurrence rates were similar. However, the study was underpowered and further larger RCTs are required.

In conclusion, the findings of this retrospective study suggest that IPOM with peritoneal bridging is as feasible and safe as conventional defect closure and simple non-defect closure techniques. In case of longstanding hernia with thinning or atrophy of the skin covering the hernia, IPOM-pb may not be preferable. Likewise, IPOM-pb may not be preferred in multiple smaller hernia that may complicate the procedure and prolong operation time. However, in view of the size and retrospective nature of this single-centre study, a large randomised controlled trial is required to compare the postoperative outcomes of these three laparoscopic IPOM approaches more accurately.

## Data Availability

Not applicable.
